# Death of Dr. Tomlinson Fort

**Published:** 1859-06

**Authors:** 


For the Atlanta Medical and Surgical Journal.

**DEATH OF DR. TOMLINSON FORT.**

The readers of this journal will learn with profound sorrow, that Dr. Tomlinson Fort, one of the most venerable and eminent members of the medical profession of Georgia, departed this life at his residence in Milledgeville, on the morning of Wednesday, the eleventh of May. Though advanced in years, he was still vigorous; and none of the brightness of his intellect had been dimmed by the waste of time. He has sunk into the grave, and left us to feel that the loss is one of no common character, either to the profession which he adorned, or the commonwealth of which he was so worthy a citizen. Half a century of activity as a physician and a statesman, has secured for Dr. Fort a name that the people "will not willingly let die."—His life furnishes much instruction to others, by showing, that honor and virtue, and intelligence, are the true standards of greatness; and that though there may be many obstacles to be overcome, by holding these as the cardinal points of life, the goal of success may be finally achieved.

Dr. Fort sprang from a worthy sire, a patriot and a statesman of the revolutionary struggle. His father, Arthur Fort, was a soldier in those times, when to be a soldier, was to imperil not only life, but property and family. Tradition has encircled his brow with enduring laurels; it has said of him, that he constituted one of the committee of Public Safety, to whose wisdom and prudence and vigilance, in those dark hours of danger and trial, the lives of their countrymen were entrusted; and it has transmitted to his posterity the gratifying assurance, that he contributed his full share in the protection of the colonies in their time of struggle, and that he aided in safely launching them into independence. He was among those who were chosen by the people, to frame a constitution for the State of Georgia, when she emerged from the revolution. He united in constructing the new government, and was one of the signers of the Organic Law of our State. Living to a green old age, he was permitted to see the country for which he had fought, and suffered and labored, advancing in a career of unexampled prosperity. His son, Tomlinson, born in the county of Warren, on the 11th of July, 1787, was brought up inured to the hardships and privations of a newly settled territory. Knowing little of his early life, we can only say, that he never had the advantages of a collegiate education. His own untiring energy; his love of learning, combined with a strong native intellect, enabled him to acquire the vast amount of knowledge of which he was the possessor. Early in the present century, he repaired to Philadelphia to attend Medical Lectures, and qualify himself for the practice of his profession. The celebrated Dr. Rush, was then in the zenith of his fame, and under the charm of his prelections, Dr. Fort first imbibed that remarkable zeal in medical science, which distinguished him throughout his long life. Soon after obtaining his degree, he located in the town of Milledgeville, and commenced the practice of medicine. Devoting himself with great ardor to his duties and studies, he soon began to occupy a high position, and this eminence he maintained for fifty years. It may justly be said of him, that few men have been regarded with greater confidence in the management of diseases.

His professional career was not the only channel through which Dr. Fort acquired his wide-spread reputation. Eminently fitted for public service he was often called on by his fellow citizens to occupy positions of responsibility and usefulness. When the Seminole Indians were harassing the frontiers of Georgia and Florida—Dr. Fort offered his services, and was appointed to a command under Gen. Daniel Newnan. During the campaign in which he served, he was wounded in the knee, so seriously, that we believe he never entirely recovered from it. The ball was not extracted until within the last few years of his life.

During the memorable struggles between Gen. Clark and George M. Troup, Dr. Fort was identified as one of the prominent leaders, of what was then called the "Clarke Party." Those who remember the fierce and furious warfare of that period, inform us that it was a contest between giants, and that no such scenes have ever been enacted in Georgia since. The struggles and the leaders have now nearly all passed away, and their feuds are spoken of without a vestige of the passion and feeling which characterized the manner in which they were conducted. New political organizations have been established, and those who once were bitter enemies, have been found standing side by side in defence of the same common principles. Dr. Fort was for a number of years, a member of the Georgia Legislature, from the county of Baldwin, and took an active part in the advocacy of all measures calculated to promote and elevate the State. So highly esteemed were his services, that in 1827 and 1828, he was elected on the general ticket, one of the Representatives from Georgia, in the congress of the United States. On his retirement from this post, he became President of the Central Bank, and continued to discharge the duties of that responsible station for a number of years. He distinguished himself as an earnest and powerful advocate of the Internal Improvement System, commenced by the State twenty-five years ago, and we have heard from a contemporary and early friend of Dr. Fort, that no man contributed more to the prosecution of our great State work—the Western & Atlantic Railroad.

The most remarkable feature of Dr. Fort's intellect, was its striking originality. He seemed to view all questions in a manner strictly his own, and his powerful mind never failed to shed new light on everything it touched. His knowledge of books was extensive and diversified. In conversation he poured out vast streams of curious lore, and seemed familiar with every subject that could be suggested. It was delightful to listen to him expatiating on some point in physical science, or in history. What was before mysterious or hardly explicable, became under his lucid and comprehensive explanation, clear and interesting. He stripped science of its difficulties, and made it accessible to the most uninitiated.

His home at Milledgeville, was, for a quarter of a century, a notable place, frequented by a wide circle of the learned and able men of the State ; of that circle he was the centre, and those who were wont to surround him in social enjoyment, will feel that there is a void which can never be filled, and that death has robbed the capital of one of its greatest attractions. Let his example never be forgotten, but brighten with accumulating years, and let his memory be sacred in the profession of which he was so bright an ornament, and among the people to whom he consecrated so much of his active life.

We append the following report of the proceedings of the physicians of Milledgeville relative to the death of Dr. Fort:

"At a meeting of the physicians of Milledgeville, held this day, Dr. G. D. Case was called to the chair, and Dr. S. G. White requested to act as secretary, when the following preamble and resolutions were adopted :

Whereas, it has pleased the Almighty to remove from his sphere of usefulness our venerable and beloved brother, Tomlinson Fort, who for nearly fifty years has labored arduously, with great ability and success professionally in this community, and whose eminent reputation is commensurate with the southern country.

*Resolved*, That we deeply deplore the loss our profession has sustained in the death of one, who for so many years, has adorned, dignified and most creditably exercised our time-honored vocation.

*Resolved*, That we sincerely sympathize with the family of our deceased brother, in this their sorest affliction, and heartily commend them to Him, who alone can medicate to the broken heart, and soothe the wounded spirit.

*Resolved*, That we will, as a body attend the funeral obsequies of our departed brother, and wear the usual badge of mourning thirty days.

*Resolved*, That the Secretary be requested to present a copy of these proceedings to the family, and furnish the papers of the city with another for publication.

*Resolved*, That we consider these resolutions merely as a professional tribute to our brother, leaving the detailed and minute recital of his history, to those who may write his obituary.

*Resolved*, That a notice of the death of our venerated brother, be published in the Medical Journals, of the State.

G. D. CASE, Chairman.

S. G. White, Secretary.
Milledgeville, May 11, 1859.

